# Increasing trends in admissions due to non-communicable diseases over 2012 to 2017: findings from three large cities in Myanmar

**DOI:** 10.1186/s41182-020-00209-8

**Published:** 2020-04-24

**Authors:** Ei Ei Swe, Kyaw Ko Ko Htet, Pruthu Thekkur, Lwin Lwin Aung, Lwin Lwin Aye, Thazin Myint

**Affiliations:** 1 Department of Medical Research (Pyin Oo Lwin Branch), Pyin Oo Lwin, Myanmar; 2 International Union Against Tuberculosis and Lung Disease (The Union), Paris, France; 3 The Union South-East Asia Office, New Delhi, India; 4 Health Information Division, Department of Public Health, Nay Pyi Taw, Myanmar; 5 Non-Communicable Diseases Control Unit, Department of Public Health, Nay Pyi Taw, Myanmar; 6 Medical Record Unit, Department of Medical Services, Nay Pyi Taw, Myanmar

**Keywords:** Disease profile, Diabetes, Hypertension, Cardiovascular diseases, Chronic respiratory diseases, ICD 10, HMIS

## Abstract

**Background:**

Globally, cardiovascular diseases, chronic respiratory diseases, cancers, and diabetes are the four major non-communicable diseases (NCDs) contributing to more than 80% of mortality and morbidity due to NCDs. In Myanmar, the proportional mortality rate due to NCDs increased from 46.9% in 2000 to 68% in 2017. However, the trends and patterns of four major NCDs or their hospital admissions are not known. In this regard, we aimed to assess the trends and profile of admissions with four major NCDs using final diagnosis coded in International Classification of Diseases-2010 version (ICD-10) from medical record data of the large tertiary hospitals in different regions of Myanmar.

**Results:**

Of the 774,970 total admissions in the study hospitals, the median and interquartile range (IQR) age was 39 (25–55) years and 51.6% were males. Over a 6-year period, there was not only 2.2-fold increase in the number of admissions due to any of four major NCDs but also their proportion increased significantly from 18.8% in 2012 to 25.4% in 2017 (chi-square for trend, *p* value < 0.001). The number of admissions due to cancers, cardiovascular diseases, and chronic respiratory diseases also showed linear increasing trends at the rate of 1741 (95% CI 766 to 2715), 1797 (95% CI 345 to 3249), and 597 (95% CI 530 to 612) per year, respectively. Though the admissions with diabetes increased over the years, the rate of increase of 284 (95% CI − 60 to 628) per year was not statistically significant. Among cancer admissions, colorectal (13.1%), breast (13.0%), and lung (11.0%) cancers were the commonest. Stroke (30.6%) and ischemic heart disease (21.9%) admissions were the highest among the cardiovascular diseases. Chronic obstructive pulmonary disease (35.5%) and type 2 diabetes (53.9%) were commonest among chronic respiratory diseases and diabetes, respectively.

**Conclusion:**

There was a disproportionate increase in NCD admissions which requires tertiary health facilities to increase their infrastructure and trained workforce to cater to such admissions. The primary health care facilities have to be strengthened for prevention, early detection, and efficient management of NCDs to prevent life-threatening complications requiring hospitalization.

## Background

Globally, non-communicable diseases (NCDs) have become the major cause of morbidity and mortality over the last three decades [1]. About 41 million people die due to NCDs each year, accounting for about 71% of all deaths [2]. The deaths due to NCDs are of greater concern as about 37% (~ 15 million) of them are premature deaths occurring in the economically productive age of 30 to 69 years [2]. The NCDs affect the low- and- middle-income countries (LMICs) disproportionately with more than three-fourth (~ 32 million) deaths due to NCDs occurring in these countries and 85% of them are premature deaths [3].

Globally, cardiovascular diseases, chronic respiratory diseases, cancers, and diabetes are the four major NCDs leading to more than 80% of mortality and morbidity [3]. The efficient management of these four major NCDs is imperative to achieve the Sustainable Development Goal (SDG) target of reducing premature deaths from NCDs by one-third by 2030 [4]. Globally, an estimated US$ 47 trillion would be required for the management of these four NCDs along with mental illness in the next two decades [5].

The early diagnosis, lifelong drug support, early detection, and effective management of complications are the mainstay in averting premature mortality due to these major NCDs. Cancers are largely treated with hospitalization at tertiary care hospitals. Though individuals with cardiovascular diseases, chronic respiratory diseases, and diabetes are managed through ambulatory care, the life-threatening complications which they often develop require hospitalization and specialist care at tertiary care hospitals. Thus, information on trends and patterns of admissions with major NCDs is important to re-orient the infrastructure and capacity of health professionals at hospitals to manage these admissions efficiently. The studies exploring trends and patterns of NCDs among inpatients using Health Information Management System (HMIS) data in China [6], Nepal [7], and Nigeria [8] showed an increase in NCD admissions over the years and also variation in pattern across age, gender, and geographic region.

Like any other LMICs, Myanmar has a high and rising burden of NCDs. The proportional mortality rate due to NCDs has increased from 46.9% of all deaths in 2000 to 68% in 2017 [9]. Acknowledging this, the Ministry of Health and Sports has identified NCDs as a priority public health problem in the National Health Policy of Myanmar (2017–2021) [10]. The program focusing on four major NCDs has been initiated [10]. One of the six objectives of Myanmar National Strategic Plan for prevention and control of NCDs (2017–2021) was to monitor the trends and determinants of NCDs and its risk factors through the establishment of sustainable surveillance and evaluation mechanisms [10]. However, there have been no longitudinal studies reporting on trends and spectrum of major NCDs or their hospital admissions. This is because of the lack of a robust HMIS system for capturing disease profiles at the community and primary health facilities.

However, the tertiary general hospitals have a Computer-Assisted Medical Record System (CAMRS) installed since 2010, capturing demographic and morbidity details of all the individuals admitted to the hospitals. The disease for which the patient was treated during the admission is coded as per the International Classification of Diseases-2010 version (ICD-10) [11]. This system provides an opportunity to elucidate the trend and pattern of hospital admissions due to NCD over the years.

Hence, in this study, among inpatient admissions in three selected tertiary hospitals located in different geographical regions of Myanmar, we aimed to (1) assess the number and proportion of admissions with any of the four major NCDs, (2) describe the trends of the number of admissions with four major NCDs over 6 years (2012 to 2017), and project the probable number of admissions in the year 2020, (3) patterns of major NCDs stratified by age and gender.

## Methods

### Study design

This was a cross-sectional descriptive study using secondary data routinely collected by the medical record department of tertiary hospitals in Myanmar.

### Study setting

#### General setting

Myanmar has a population of 51.5 million. Nay Pyi Taw is the capital of Myanmar. Yangon and Mandalay are the largest and second-largest regions with about one-fourth of the total population of the country residing in these regions [12]

##### Health care service delivery in Myanmar

In Myanmar, the public health sector has a three-tier system with primary, secondary, and tertiary health facilities [13]. The tertiary facilities with the specialist physician, well-established laboratories, and inpatient wards provide healthcare services to patients with all forms of diseases requiring specialist care. The number of primary, secondary, and tertiary health facilities all over the country is 1808, 803, and 75, respectively.

#### Specific setting

This study was conducted in the three largest tertiary general hospitals with a high bed occupancy rate and representing different regions of Myanmar. The North Okkalapa General Hospital (800 bedded) of Yangon situated in the lower region of Myanmar, Mandalay General Hospital (1500 bedded) situated in the central region, and Nay Pyi Taw General Hospital (1000 bedded) located in the Nay Pyi Taw Union Territory were included. Though Yangon General Hospital was the largest tertiary hospital in the lower region of Myanmar, we included North Okkalapa General Hospital due to the non-availability of electronic data and administrative approval for accessing data in the former.

All three hospitals have both medical and surgical departments. Mandalay General Hospital has no separate obstetrics and gynecology and pediatrics wards. However, it has an oncology ward where even the gynecological and pediatric cancer patients are admitted for chemotherapy and radiation services. Patients reaching these hospitals are either referred from primary and/or secondary level facilities or walk-in directly into the tertiary health facilities. The patients are examined and admitted by the assigned physician based on the severity of the disease.

##### Coding of ICD-10 and reporting

On admission, details of the patients are recorded in the “inpatient book” and “patient chart” with a unique ID number. When the patient is discharged or died, the patient chart is collected at the hospital. In the patient chart, the treating physician documents the final diagnosis. All the patient charts are sent to the medical record department (MRD) and are archived in the department.

The technical advisors of MRD code the final diagnosis, according to ICD-10. The trained data entry operator in MRD enters the individual patients’ details like age, sex, date of admission, date of discharge, ICD-10 code, and type of discharge into the CAMRS maintained by HMIS unit of Department of Public Health (DPH).

The data entry operator downloads the CAMRS data into compact-disk (CD) and sends the CD to the HMIS unit at Nay Pyi Taw every year. At the HMIS unit, these annual data files from each of these hospitals are compiled and prepared in Microsoft Excel format.

### Study population

All admissions in North Okkalapa General Hospital, Mandalay General Hospital, and Nay Pyi Taw General Hospital from 1 January 2012 to 31 December 2017 were included in the study.

### Data variables, sources of data, and data collection

The details like age in completed years, sex, dates of admission, hospital, and ICD code of final diagnosis were extracted from the HMIS database obtained from DPH. The principal investigator obtained the data files in Microsoft Excel from 2012 to 2017 during the month of April 2019.

The admissions with ICD-10 code of final diagnosis corresponding to four major NCDs like malignant neoplasms (cancer), chronic respiratory diseases, cardiovascular diseases, and diabetes were grouped under each disease category. Within the major NCDs, each of the ICD-10 codes was labeled with the condition they represent. The classification used by the Global Burden of Diseases (GBD) was used to categorize the diseases (Supplementary Table 1). The common malignant neoplasms, cardiovascular diseases, chronic respiratory diseases, and diabetes-related admissions were used as outcome variables that were described across gender, age, and year of admission.

### Data analysis and statistics

Data obtained in the Microsoft Excel format was analyzed using EpiData analysis software (version 2.2.2.182 EpiData Association Demark).

The frequency and proportion of inpatient admissions with the final diagnosis of any of the four NCDs were calculated across the year of admission, hospital, age groups, and gender. The chi-squared test for trend was used to assess the increase or decrease of proportions of NCD admissions to the total over the study period.

The line graphs depicting trend in the number and forward projections using linear regression were developed for admissions with any of the four major NCDs. Similarly, the trend in the number and forward projections was developed for admissions with any of the four major NCDs in each of the study hospitals. The forward projections for the year 2020 were made forecasting using linear regression on Microsoft Excel 2016. The beta-coefficients (linear rate of increase) with 95% CI were reported for each model developed.

The frequencies of the ten most common cancers and cardiovascular diseases, chronic respiratory diseases, and diabetes were described across the year of admission, age groups, and gender.

## Results

### Overall admissions and trends in admission with four major NCDs

During the study reference period, there were 774,970 admissions in three hospitals. The median and interquartile range (IQR) age of those admitted was 39 (25–55) years and 51.6% were males. Of the total, 45.2% were admitted in Mandalay General Hospital, 32.6% in North Okkalapa General Hospital of Yangon, and 22.3% in Nay Pyi Taw General Hospital (Table 1).

**Table 1:** Number and proportion admissions with at least one major NCD stratified by age, gender, and year of admission in three tertiary hospitals of Myanmar during 2012–2017

Variable	Categories	Overall admissions	Admission due to NCDs	
			***n***	(%)*
**Total**		**774,970**	**169,197**	**(21.8)**
**Age in years**				
	< 5	41,752	792	(1.9)
	5–14	35,287	2029	(5.7)
	15–24	114,664	7312	(6.4)
	25–34	139,319	13,050	(9.4)
	35–44	121,847	22,966	(18.8)
	45–54	115,566	37,321	(32.3)
	55–64	100,139	40,695	(40.6)
	65–74	66,548	28,699	(43.1)
	75–84	32,604	13,557	(41.6)
	≥ 85	7244	2776	(38.3)
**Gender**				
	Male	399,985	81,991	(20.5)
	Female	374,985	87,206	(23.3)
**Year of admission**				
	2012	94,351	17,775	(18.8)
	2013	111,411	22,958	(20.6)
	2014	120,228	23,649	(19.7)
	2015	140,780	28,615	(20.3)
	2016	152,587	36,650	(24.0)
	2017	155,613	39,550	(25.4)
**Hospital**				
	Mandalay	349,953	98,216	(28.1)
	Yangon	252,490	40,794	(16.2)
	Nay Pyi Taw	172,527	30,187	(17.5)

*Row percentage

Of the total, 21.8% were admitted due to any major NCDs. The proportion was 28.1% in Mandalay General Hospital, 16.2% in North Okkalapa General Hospital of Yangon, and 17.5% in Nay Pyi Taw General Hospital.

In 2012, the proportion with any major NCDs among all the admissions was 18.8% whereas in 2017, it was 25.4%. There was a statistically significant increasing trend in the proportion of admissions due to any of the four NCDs over 2012 to 2017 (chi-square for trend, *p* value = 0.0027) (Table 1).

In total, there was a 2.2-fold increase in the number of admissions due to any of the major NCDs from 17,775 in 2012 to 39,550 in 2017. Over the same period, the number of admissions due to major NCDs increased by 1.7 times in Mandalay General Hospital, 3.4 times in North Okkalapa General Hospital, and 2.5 times in Nay Pyi Taw General Hospital.

**Fig. 1:**
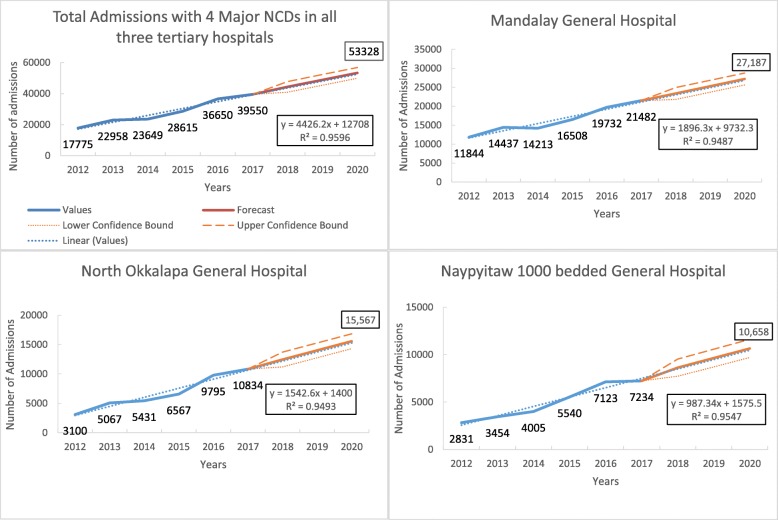
Trends in the number of admissions with major NCDs and within three tertiary hospitals of Myanmar during 2012–2017

Over 6 years, the number of admissions due to NCDs showed a linear increasing trend at the rate of 4426 (95% CI 2885 to 5954, *p* value = 0.0013) per year. With the same linear increasing trend, it is estimated that by 2020, there will be about 53,328 admissions due to NCDs. The average rate of increase per year was 1896 (95% CI 1184 to 2607, *p* value = 0.0017) in Mandalay General Hospital, 1542 (95% CI 946 to 2131, *p* value = 0.0019) in North Okkalapa General Hospital, and 987 (95% CI 640 to 1328, *p* value = 0.0013) in Nay Pyi Taw General Hospital (Fig. 1).

### Trends and pattern of individual NCDs

#### Malignant neoplasms

**Fig. 2:**
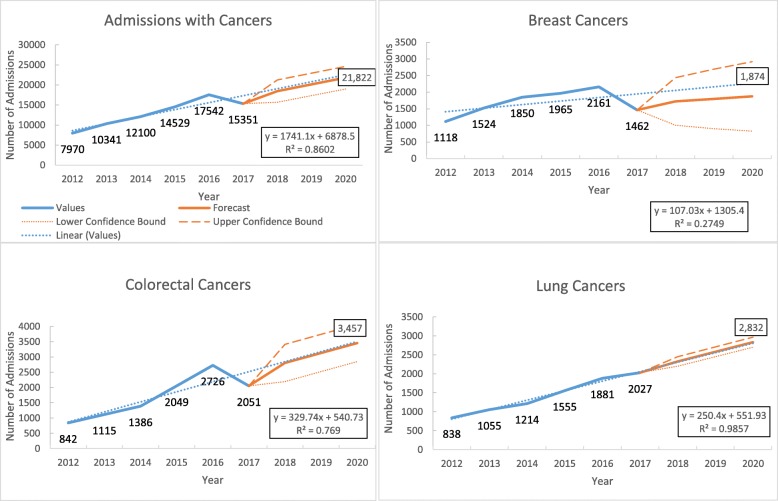
Trends in the number of admissions with common cancers in three tertiary hospitals of Myanmar during 2012–2017

Of the total admissions, about 10.0% of the admissions were due to malignant neoplasms (data not shown in the figure). The number of admission due to malignant neoplasms increased from 7970 to 15,351 during 2012–2017 with a linear increasing trend at the rate of 1741 (95% CI 766 to 2715, *p* value = 0.0167) per year (Fig. 2).

Among the malignant neoplasms, colorectal cancer (13.1%), breast cancer, and lung cancer were the commonest. Over 6 years, the admissions due to colorectal cancer increased at the rate of 329 (95% CI 78 to 580, *p* value = 0.017) per year, breast cancer increased at the rate of 107 (95% CI − 134 to 348, *p* value = 0.419) per year, and lung cancer increased at the rate of 250 (95% CI 208 to 292, *p* value = 0.002) per year (Fig. 2 and Supplementary Table 2).

Of all the malignant neoplasms, the most common neoplasms among males were lung cancer (15.4%), liver cancer (14.8%), and colorectal cancer (13.6%) whereas in females, it was breast cancer (23.4%), colorectal cancer (12.7%), and liver cancer (10.2%). In those aged less than < 15 years and aged between 15 and 29 years, the most common cancer was leukemia, lymphoma, and bone and connective tissue cancer (Tables 2 and 3).

**Table 2:** Common malignant neoplasms, cardiovascular diseases, chronic respiratory diseases, and diabetes-related admissions stratified by gender in three tertiary hospitals of Myanmar during 2012–2017

	Admission due to disease					
Category	Male			Female		
		*n*	(%)*		*n*	(%)*
**Malignant neoplasams**^**#**^		**34,959**	**(100.0)**		**42,874**	**(100.0)**
	Lung cancer	5398	(15.4)	Breast cancer	10,034	(23.4)
	Liver cancer	5160	(14.8)	Colorectal cancer	5426	(12.7)
	Colorectal cancer	4743	(13.6)	Cervix cancer	4475	(10.4)
	Stomach cancer	3150	(9.0)	Lung cancer	3172	(7.4)
	Leukemia	2616	(7.5)	Leukemia	2774	(6.5)
**Cardiovascular diseases**^**#**^		**30,988**	**(100.0)**		**27,066**	**(100.0)**
	Stroke	10,721	(34.6)	Stroke	7045	(26.0)
	Other cardiovascular disease	7394	(23.9)	Other cardiovascular disease	6259	(23.1)
	Ischemic heart disease	6455	(20.8)	Ischemic heart disease	6232	(23.0)
	Inflammatory heart disease	2015	(6.5)	Rheumatic heart disease	3397	(12.6)
	Rheumatic heart disease	1859	(6.0)	Hypertensive heart disease	1929	(7.1)
**Chronic respiratory diseases**		**12,668**	**(100.0)**		**10,933**	**(100.0)**
	Other chronic respiratory diseases	7550	(59.6)	Other chronic respiratory diseases	5230	(47.8)
	Chronic obstructive pulmonary disease	4197	(33.1)	Chronic obstructive pulmonary disease	4172	(38.2)
	Asthma	921	(7.3)	Asthma	1531	(14.0)
**Diabetes**		**3376**	**(100.0)**		**6333**	**(100.0)**
	Type 1 diabetes	75	(2.2)	Type 1 diabetes	75	(1.1)
	Type 2 diabetes	1694	(50.2)	Type 2 diabetes	3540	(55.9)
	Other diabetes mellitus	1607	(47.6)	Other diabetes mellitus	2718	(42.9)

*Column percentage

^#^Only five common diseases under the category are listed and frequency will not add to the total

**Table 3:** Most common admissions with major NCDs stratified by age in three tertiary hospitals of Myanmar during 2012–2017

Age in years	Malignant neoplasms			Cardiovascular diseases			Chronic respiratory diseases			Diabetes mellitus (DM)		
		*n*	%		*n*	%		*n*	%		*n*	%
**< 15**	**Total**	**846**		**Total**	**724**		**Total**	**12,088**		**Total**	**43**	
	Leukemia	319	(37.7)	Rheumatic	214	(29.6)	Others	634	(52.5)	Other DM	26	(60.5)
	Lymphoma	165	(19.5)	Stroke	126	(17.4)	Asthma	481	(39.8)	Type 2	10	(23.3)
	B and T*	120	(14.2)	Non-rheumatic valvular	103	(14.2)	COPD	93	(7.7)	Type 1	7	(16.3)
**15–29**	**Total**	**5796**		**Total**	**4184**		**Total**	**2480**		**Total**	**412**	
	Leukemia	1703	(29.4)	Rheumatic	939	(22.4)	Others	1968	(79.0)	Other DM	232	(56.3)
	Lymphoma	832	(14.4)	Stroke	609	(14.6)	Asthma	363	(14.6)	Type 2	126	(30.6)
	B and T	428	(7.4)	Inflammatory	469	(11.2)	COPD	149	(6.0)	Type 1	54	(13.1)
**30–44**	**Total**	**15,108**		**Total**	**10,152**		**Total**	**3685**		**Total**	**1511**	
	Breast	2574	(17.0)	Stroke	2465	(24.3)	Others	2890	(78.4)	Type 2	750	(49.6)
	Colorectal	2548	(16.9)	Rheumatic	1629	(16.0)	Asthma	505	(13.7)	Other DM	703	(46.5)
	Liver	1601	(10.6)	Ischemic	1078	(10.6)	COPD	290	(7.9)	Type 1	58	(3.8)
**45–59**	**Total**	**30,432**		**Total**	**18,680**		**Total**	**5035**		**Total**	**4250**	
	Breast	5361	(17.6)	Stroke	6088	(32.6)	Others	3249	(64.5)	Type 2	2358	(55.5)
	Colorectal	3449	(11.3)	Ishemic	4213	(22.6)	COPD	1153	(22.9)	Other DM	1872	(44.0)
	Liver	3122	(10.2)	Rheumatic	1657	(8.9)	Asthma	633	(12.6)	Type 1	20	(0.5)
**≥ 60**	**Total**	**25,651**		**Total**	**24,314**		**Total**	**11,193**		**Total**	**3493**	
	Lung	5102	(19.9)	Stroke	8478	(34.9)	COPD	6684	(59.7)	Type 2	1990	(57.0)
	Colorectal	3254	(12.7)	Ishemic	7245	(29.8)	Others	4039	(36.1)	Other DM	1492	(42.7)
	Liver	2477	(9.7)	Hypertensive	1499	(6.2)	Asthma	470	(4.2)	Type 1	11	(0.3)

*Bone and connective tissue

#### Cardiovascular diseases

**Fig. 3:**
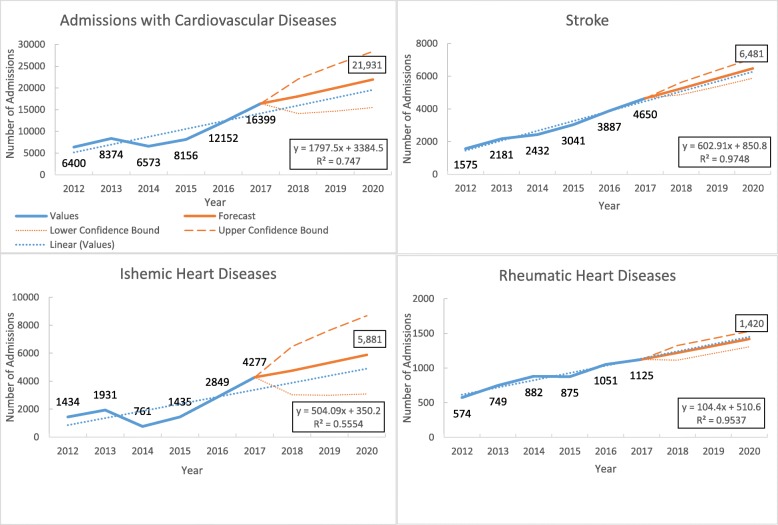
Trends in the number of admissions with common cardiovascular diseases in three tertiary hospitals of Myanmar during 2012–2017

Of the total admissions, 7.4% were admitted with cardiovascular diseases (data not shown in the figure). There was an increase in the number of patients admitted with cardiovascular disease from 6400 in 2012 to 16,399 in 2017, with a linear increasing trend at the rate of 1797 (95% CI 345 to 3249, *p* value = 0.05) per year (Fig. 3).

Among the cardiovascular diseases, the commonest were stroke and ischemic heart disease. Over 6 years, the admissions due to stroke increased at the rate of 602 (95% CI 468 to 737, *p* value = 0.002) per year and ischemic heart disease increased at the rate of 504 (95% CI − 122 to 1130, *p* value = 0.136) per year (Fig. 3 and Supplementary Table 3).

Of all the cardiovascular diseases, the most common diseases among both males and females were stroke (34.6% and 26.0%) and ischemic heart diseases (20.8% and 23.0%). Among admissions of those aged less than < 15 years and aged between 15 and 29 years, the rheumatic heart disease (29.6% and 22.4%) was the commonest. In those aged more than 30 years, stroke (24.3%) is the most common cause of admission due to cardiovascular diseases (Tables 2 and 3).

#### Chronic respiratory diseases

**Fig. 4:**
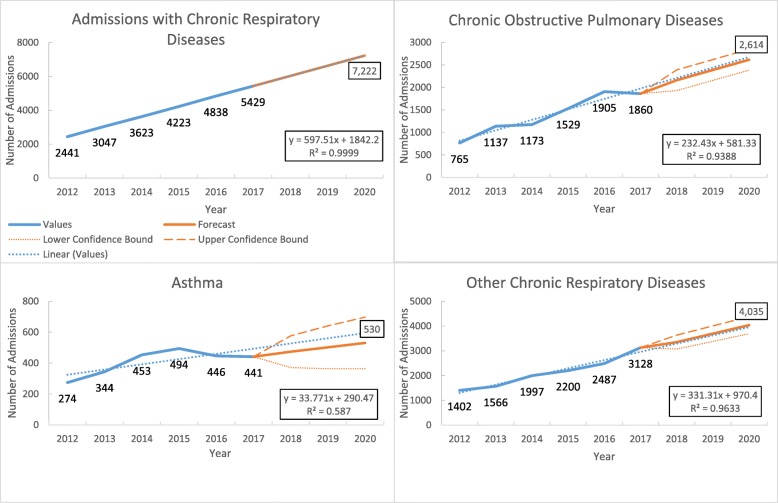
Trends in the number of admissions with common chronic respiratory diseases in three tertiary hospitals of Myanmar during 2012–2017

Of the total admissions, 3% were admitted with chronic respiratory diseases (data not shown in the figure). The admissions due to chronic respiratory diseases increased from 2441 in 2012 to 5249 in 2017, with a linear increasing trend at the rate of 597 (95% CI 530 to 612, *p* value < 0.001) per year (Fig. 4).

Among all admitted chronic respiratory diseases, chronic obstructive pulmonary disease and asthma were common diseases. Over 6 years, the annual admissions due to chronic obstructive pulmonary disease increased at the rate of 232 (95% CI 150 to 314, *p* value = 0.016) and asthma increased at the rate of 33 (95% CI − 5.5 to 73, *p* value = 0.355) (Fig. 4 and Supplementary Table 4).

#### Diabetes

**Fig. 5:**
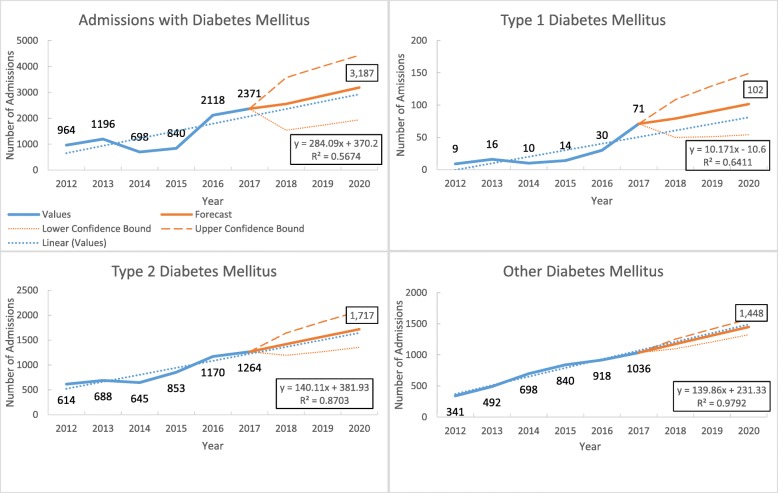
Trends in the number of admissions with diabetes mellitus in three tertiary hospitals of Myanmar during 2012–2017

Of the total admissions, diabetes accounted for 1.2% (data not shown in the figure). Admission with diabetes was showing increasing from 964 to 2371 between 2012 and 2017. There was no significant linear increasing trend with the rate of 284 (95% CI − 60 to 628, *p* value = 0.297) per year (Fig. 5).

The proportion of type 2 diabetes was 50.2% in male and 55.9% in female. Over 6 years, average annual admissions due to type 1 diabetes increased at the rate of 10 (95% CI − 0.3 to 20, *p* value = 0.055) and type 2 diabetes increased at the rate of 140 per year (95% CI 65 to 215, *p* value = 0.016) (Fig. 5 and Supplementary Table 5).

## Discussion

We conducted this study to elucidate the trends over 6 years (2012–2017) and patterns of four major NCDs among inpatient admissions at three selected tertiary care hospitals of Myanmar. The study has five key findings. First, there was a significant increase in the number and proportion of inpatient admissions due to any of the four major NCDs over 6 years. Second, there was about a twofold increase in inpatient admissions due to malignant neoplasms, cardiovascular diseases, chronic respiratory diseases, and diabetes. Third, lung cancer and liver cancer were the commonest cancer among males and showed an increasing trend over the years. Fourth, about one out of three inpatient admissions due to cardiovascular disease were due to stroke with a threefold increase over 6 years. Fifth, though the number of admissions due to diabetes increased over the years, the rate of increase was not statistically significant.

The study has some strengths. We used routinely collected HMIS data in which ICD-10 coding was consistently used in all three hospitals during our study reference period and, thus, limited the potential misclassifications due to changes in the coding system. Also, the classification of diseases based on the widely used ICD-10 coding system enhanced the validity and comparability of the study findings globally. We included three hospitals with high bed occupancy rates and representing different regions of the country. Thus, it could have accounted for regional variations in the pattern of inpatient admissions. However, the generalizability is limited as only one tertiary hospital each from the three regions was included. We adhered to Strengthening the Reporting of Observational studies in Epidemiology (STROBE) for reporting the study findings.

However, the study also has a few limitations. First, we performed trend analysis with the number of admissions as unit of analysis as there was no unique identifier to aggregate the admissions at the patient level. As we ended up counting each patient admission as disease count, we could have over-represented some of the conditions like malignant neoplasms, which require multiple hospital admissions for treatment. Second, only the cause of patient admissions was captured in the HMIS and the comorbidities were not documented. For example, the inpatient admissions with stroke might have had comorbid conditions like diabetes and/or hypertension but were coded only with ICD-10 code related to stroke. This meant diabetes and hypertension were coded only when these were the primary cause and diagnosis for admission. Thus, comorbid conditions like diabetes and hypertension, which could cause life-threatening complications, might have been under-represented. Third, as one of the facilities did not have a gynecology department, the cancers specific to female gender like cervical cancer, uterine cancer, and ovarian cancers might have been under-represented. This might also be the reason for admissions with breast cancer being commonest in our study contrary to findings from community-based studies showing cervical cancers to be commonest. We failed to include specialized women’s tertiary hospitals in Yangon and Mandalay due to difficulties in the acquisition of complete data for the study years. Fifth, our study was a facility-based study and might not reflect the trends and patterns of diseases in the community**.**

Though overall admissions increased by 1.6 times during the study period, the admissions with any four major NCDs increased by 2.2 times. The proportion of admissions with any four major NCDs also increased over the years in all the three hospitals. By 2017, about one-fourth of admissions were due to those major NCDs. A study conducted in a tertiary teaching hospital of Nepal using ICD-10 data reported 30% inpatient admissions due to alcohol-related major NCDs [14]. Also, a study conducted in China across 12 tertiary hospitals showed an increase in the number of admissions due to NCDs from 110,796 in 2003 to 234,876 in 2014 [6]. These findings from other studies substantiate our inference of the increasing burden of NCD among inpatient admissions [15, 16].

The increase in admissions with NCD in Myanmar might be due to several reasons listed here. First, unhealthy lifestyle behaviors could have contributed to an increase in NCDs [2, 17, 18]. According to the 2014 STEPs survey report, among adults, one-fifth had tobacco consumption, two-third had alcohol consumption, and a few (2.5%) had fruit and vegetable consumption [19]. The tobacco use, alcohol use, and low fiber diet are known risk factors of lung cancer, liver cancer, and colorectal cancers [20], which were the three most common cancers among males in the current study. Similarly, tobacco use is associated with the occurrence and exacerbation of chronic obstructive pulmonary disease [21], which also showed a threefold increase in the number of admissions.

Second, late detection and deficiencies in the comprehensive management of diabetes and/or hypertension at primary healthcare level might have led to life-threatening complications requiring hospital admissions and management [22]. For example, the complications of hypertension and/or diabetes like stroke have had a steep increase during the study period. The increase could have been averted if all individuals with hypertension and diabetes were detected early and their blood pressure and blood sugars were maintained below the target level through appropriate management at primary health facilities [23]. The major reason for late diagnosis and treatment in Myanmar was the lack of trained health staff for managing NCDs at primary health facilities [24]. Overall there is a shortage of doctors with Myanmar’s human resources for health in 2017 reporting that 13 out of 15 states and regions were below the WHO recommended minimum number of 1 per 1000 population for medical doctor [25]. Also, a study assessing the implementation of NCD care at primary health facilities of Myanmar reported a lack of infrastructure and drugs for managing NCD patients at primary health facilities [24].

Third, though it might not reason out the disproportionate increase in admissions with any of the four major NCDs, the increase in government funding and free of charge health care services could have increased the overall utilization of services in tertiary health facilities [26]. There was a ninefold increase in health funding from 2010 to 2017 (from 94 million US$ in 2010–2011 to 850 million US$ in 2016–2017), which was mainly used to finance medical care in hospital settings [26]. The increased financial investment could have improved the infrastructure and would have increased the number of admissions. Also, the improved transportation has made it easier for people living in rural areas to seek care from well-equipped and specialized tertiary hospitals in big cities [27]. As there is no referral policy in place, the patients with relatively less severe diseases or complications walk-in and request admission directly at a tertiary hospital, causing overburden to the hospital [27].

The study has some implications for improving healthcare services. The increasing trends of inpatient admissions due to NCDs require reorienting the tertiary health facilities to cater to the needs of such patients. The patients admitted due to cancers, cardiovascular diseases, and diabetes usually have extended duration of stay in the hospitals. The multi-modality treatments require such patients to utilize operation theaters (OT) and intensive care units (ICUs). This requires hospitals to increase the infrastructure and workforce to cater to the demand. Individuals with cancer on chemotherapy and diabetes are more prone to opportunistic infections and thus can acquire nosocomial infections. This might require strengthening infection control mechanisms or establishing separate hospitals for individuals with cancer or diabetes. As these NCDs can cause pain, physical stress, and mental stress for a longer duration, there would a need for specialized medical and nursing care to ensure good end-of-life care. This might require establishing hospital and community-based palliative care programs [28].

The better alternative for public health programs would be to limit the NCDs and complications due to NCDs, which require hospitalization at the tertiary level. The mass campaigns with a multi-media mix need to be launched, highlighting the ill effects of behavioral risk factors and increasing trends of NCDs in the country. The piloted “WHO Package of Essential Non-communicable Disease Interventions (WHO PEN)” for primary care has to be universalized throughout the country. The trained health workforce has to be increased as the country has 1.3 health workers per 1000 population, compared with WHO’s recommended threshold of 4.45 per 1000 population required for Universal Health Coverage [25]. The primary health centers need to be strengthened and community health workers have to be trained to identify, diagnose, and treat common NCDs in the community. Trained medical doctors have to be deployed in secondary hospitals to detect and manage early complications associated with diabetes and hypertension.

The ICD-10 coding of inpatient admissions at health facilities could be used for sentinel surveillance of common diseases based on long-term trends and patterns in admissions. However, there is a need for developing electronic health records (EHR) with a unique identifier assigned to each patient. Developing such an EHR could help in summarizing the disease profile with the patient as unit of analysis.

## Conclusion

There was a disproportionate increase in NCD admissions, which requires tertiary health facilities to increase their infrastructure and trained workforce to cater to such admissions. The primary health care facilities have to be strengthened for prevention, early detection, and efficient management of NCDs in order to prevent life-threatening complications requiring hospitalization.

## Supplementary information

**Additional file 1: Supplementary Table 1.** ICD-10 codes of four major non-communicable diseases- malignant neoplasms, cardiovascular diseases, chronic respiratory diseases and diabetes during 2018.

**Additional file 2: Supplementary Table 2.** Distribution of number of admissions of fifteen most common cancers during 2012 to 2017 in three tertiary hospitals of Myanmar.

**Additional file 3: Supplementary Table 3.** Distribution of number of admissions of cardiovascular diseases during 2012 to 2017 in selected tertiary hospitals of Myanmar.

**Additional file 4: Supplementary Table 4.** Distribution of number of admissions of chronic respiratory diseases during 2012 to 2017 in three tertiary hospitals of Myanmar.

**Additional file 5: Supplementary Table 5.** Distribution of number of admissions of diabetes during 2012 to 2017 in three tertiary hospitals of Myanmar.

## Data Availability

The datasets used and/or analyzed during the current study are available from the corresponding author on reasonable request.

## Abbreviations

NCDs: Non-communicable diseases
WHO: World Health Organization
ICD: International Classification of Diseases
DM: Diabetes mellitus
MOHS: Ministry of Health and Sports
HMIS: Health Management Information System
CAMRS: Computer-Assisted Medical Record System
