# Supplementary material for: Increasing trends in admissions due to non-communicable diseases over 2012 to 2017: findings from three large cities in Myanmar
Source: Trop Med Health. 2020 Apr 24;48:24. doi: 10.1186/s41182-020-00209-8 (PMC7181486; doi:10.1186/s41182-020-00209-8)
Supplement: Supplementary file 1 — Additional file 1: Supplementary Table 1. ICD-10 codes of four major non-communicable diseases- malignant neoplasms, cardiovascular diseases, chronic respiratory diseases and diabetes during 2018. [file 41182_2020_209_MOESM1_ESM.docx]

**Supplementary Table 1:** ICD-10 codes of four major non-communicable diseases- malignant neoplasms, cardiovascular diseases, chronic respiratory diseases and diabetes during 2018

| **Disease category** | **ICD 10 Code** |
| --- | --- |
| **Malignant neoplasms** |  |
| Mouth and oropharynx cancers | C00.0-14.8 |
| Oesophagus cancer | C15.0-15.9 |
| Stomach cancer | C16.0-16.9 |
| Colorectal cancer | C18.0-21.8 |
| Liver cancer | C22.0-22.9 |
| Gall bladder cancer | C23-24.9 |
| Pancreas cancer | C25.0-25.9 |
| Lung cancer | C33-34.9 |
| Bone and connective tissue cancers | C40.0-41.9,  C49.0-49.9 |
| Melanoma | C43.0-43.9 |
| Non-melanoma skin cancers | C44.0-44.9 |
| Breast cancer | C50.0-50.9 |
| Cervix cancer | C53.0-53.9 |
| Uterus cancer | C54.0-54.9, C55 |
| Ovary cancer | C56 |
| Prostate cancer | C61 |
| Testicular cancer | C62.0-62.9 |
| Bladder cancer | C67.0-67.9 |
| Kidney cancer | C64-66,  C68.0-68.9 |
| Brain cancer | C71.0-71.9 |
| Thyroid cancer | C73 |
| Lymphoma | C81.0-85.9  C96.0-96.9 |
| Multiple myeloma + other immune-proliferative cancers | C88.0-88.91, C90.0-90.2 |
| Leukemia | C91.0-95.91 |
| Larynx | C32.0-32.9 |
| Other malignant neoplasms | C17.0-17.9, C26.0-31.9, C37-39.9, C45.0-48.8, C51-52, C57.0-60.9, C63.0-63.9, C69.0-70.9, C72.0-72.9, C74.0-75.9, C76-80 |
| **Diabetes mellitus** |  |
| Type 1 diabetes | E10.0-10.91 |
| Type 2 diabetes | E11.0-11.91 |
| Other diabetes mellitus | E12.0-14.91 |
| **Cardiovascular disease** |  |
| Rheumatic heart disease | I00-09.9 |
| Ischaemic heart disease | I20.0-24.9, I25.3-25.9 |
| Stroke | I60.0-69.8 |
| Inflammatory heart disease | I30.0-33.9, I40.0-42.9 |
| Hypertensive heart disease | I10-11.9, I13.0-15.9 |
| Non-rheumatic valvular disease | I34.0-39.8 |
| Aortic aneurysm | I71.0-71.9 |
| Peripheral arterial disease | I73.0-74.9 |
| Other cardiovascular disease | I26.0-26.9, I27.1, I28.0-28.9, I43.0-45.9, I47.0-47.1, I47.9-49.9, I51.0-51.4, I52.0-52.8, I70.0-70.8, I72.0-72.9, I77-99 |
| **Chronic respiratory disease** |  |
| Chronic obstructive pulmonary disease | I27.0, I27.8-27.9, J40-44.9 |
| Asthma | J45-46 |
| Other chronic respiratory diseases | J30.0-39.9, J47, J60-99.8 |
