# Supplementary material for: Increasing trends in admissions due to non-communicable diseases over 2012 to 2017: findings from three large cities in Myanmar
Source: Trop Med Health. 2020 Apr 24;48:24. doi: 10.1186/s41182-020-00209-8 (PMC7181486; doi:10.1186/s41182-020-00209-8)
Supplement: Supplementary file 2 — Additional file 2: Supplementary Table 2. Distribution of number of admissions of fifteen most common cancers during 2012 to 2017 in three tertiary hospitals of Myanmar. [file 41182_2020_209_MOESM2_ESM.docx]

**Supplementary Table 2:** Distribution of number of admissions of fifteen most common cancers during 2012 to 2017 in three tertiary hospitals of Myanmar

|  | **Overall** | | **2012** | | **2013** | | **2014** | | **2015** | | **2016** | | **2017** | |
| --- | --- | --- | --- | --- | --- | --- | --- | --- | --- | --- | --- | --- | --- | --- |
| **Malignant Neoplasms** | **N** | **(%)** | **N** | **(%)** | **N** | **(%)** | **N** | **(%)** | **N** | **(%)** | **N** | **(%)** | **N** | **(%)** |
| Colorectal cancer | 10169 | (13.1) | 842 | (10.6) | 1115 | (10.8) | 1386 | (11.5) | 2049 | (14.1) | 2726 | (15.5) | 2051 | (13.4) |
| Breast cancer | 10080 | (13.0) | 1118 | (14.0) | 1524 | (14.7) | 1850 | (15.3) | 1965 | (13.5) | 2161 | (12.3) | 1462 | (9.5) |
| Lung cancer | 8570 | (11.0) | 838 | (10.5) | 1055 | (10.2) | 1214 | (10.0) | 1555 | (10.7) | 1881 | (10.7) | 2027 | (13.2) |
| Liver cancer | 7476 | (9.6) | 686 | (8.6) | 902 | (8.7) | 965 | (8.0) | 1336 | (9.2) | 1626 | (9.3) | 1961 | (12.8) |
| Stomach cancer | 5875 | (7.5) | 596 | (7.5) | 679 | (6.6) | 829 | (6.9) | 1100 | (7.6) | 1422 | (8.1) | 1249 | (8.1) |
| Leukemia | 5390 | (6.9) | 447 | (5.6) | 766 | (7.4) | 982 | (8.1) | 996 | (6.9) | 1206 | (6.9) | 993 | (6.5) |
| Cervix cancer | 4476 | (5.8) | 704 | (8.8) | 783 | (7.6) | 780 | (6.4) | 711 | (4.9) | 763 | (4.3) | 735 | (4.8) |
| Lymphoma | 3945 | (5.1) | 375 | (4.7) | 514 | (5.0) | 677 | (5.6) | 670 | (4.6) | 947 | (5.4) | 762 | (5.0) |
| Mouth and Oropharynx cancer | 2987 | (3.8) | 379 | (4.8) | 487 | (4.7) | 492 | (4.1) | 533 | (3.7) | 598 | (3.4) | 498 | (3.2) |
| Ovary cancer | 2546 | (3.3) | 221 | (2.8) | 408 | (3.9) | 424 | (3.5) | 493 | (3.4) | 614 | (3.5) | 386 | (2.5) |
| Oesophagaus | 2484 | (3.2) | 269 | (3.4) | 334 | (3.2) | 364 | (3.0) | 434 | (3.0) | 550 | (3.1) | 533 | (3.5) |
| Bone and connective tissue | 1754 | (2.3) | 176 | (2.2) | 193 | (1.9) | 310 | (2.6) | 343 | (2.4) | 408 | (2.3) | 324 | (2.1) |
| Larynx | 1299 | (1.7) | 197 | (2.5) | 192 | (1.9) | 170 | (1.4) | 230 | (1.6) | 258 | (1.5) | 252 | (1.6) |
| Non melanoma | 897 | (1.2) | 74 | (0.9) | 113 | (1.1) | 212 | (1.8) | 163 | (1.1) | 216 | (1.2) | 119 | (0.8) |
| Others | 9885 | (12.7) | 1048 | (13.1) | 1276 | (12.3) | 1445 | (11.9) | 1951 | (13.4) | 2166 | (12.3) | 1999 | (13.0) |
| **Total** | **77833** | **(100.0)** | **7970** | **(100.0)** | **10341** | **(100.0)** | **12100** | **(100.0)** | **14529** | **(100.0)** | **17542** | **(100.0)** | **15351** | **(100.0)** |
