# Supplementary material for: Increasing trends in admissions due to non-communicable diseases over 2012 to 2017: findings from three large cities in Myanmar
Source: Trop Med Health. 2020 Apr 24;48:24. doi: 10.1186/s41182-020-00209-8 (PMC7181486; doi:10.1186/s41182-020-00209-8)
Supplement: Supplementary file 3 — Additional file 3: Supplementary Table 3. Distribution of number of admissions of cardiovascular diseases during 2012 to 2017 in selected tertiary hospitals of Myanmar. [file 41182_2020_209_MOESM3_ESM.docx]

**Supplementary Table 3:** Distribution of number of admissions of cardiovascular diseases during 2012 to 2017 in selected tertiary hospitals of Myanmar

|  | **Overall** | | **2012** | | **2013** | | **2014** | | **2015** | | **2016** | | **2017** | |
| --- | --- | --- | --- | --- | --- | --- | --- | --- | --- | --- | --- | --- | --- | --- |
| **Cardiovascular disease** | **n** | **%** | **n** | **%** | **n** | **%** | **n** | **%** | **n** | **%** | **n** | **%** | **n** | **%** |
| Stroke | 17766 | (30.6) | 1575 | (24.6) | 2181 | (26.0) | 2432 | (37.0) | 3041 | (37.3) | 3887 | (32.0) | 4650 | (28.4) |
| Ischemic heart disease | 12687 | (21.9) | 1434 | (22.4) | 1931 | (23.1) | 761 | (11.6) | 1435 | (17.6) | 2849 | (23.4) | 4277 | (26.1) |
| Rheumatic Heart Disease | 5256 | (9.1) | 574 | (9.0) | 749 | (8.9) | 882 | (13.4) | 875 | (10.7) | 1051 | (8.6) | 1125 | (6.9) |
| Hypertensive heart disease | 3404 | (5.9) | 688 | (10.8) | 672 | (8.0) | 62 | (0.9) | 39 | (0.5) | 958 | (7.9) | 985 | (6.0) |
| Inflammatory heart disease | 3241 | (5.6) | 484 | (7.6) | 698 | (8.3) | 342 | (5.2) | 466 | (5.7) | 521 | (4.3) | 730 | (4.5) |
| Non-rheumatic valvular disease | 1407 | (2.4) | 163 | (2.5) | 195 | (2.3) | 179 | (2.7) | 187 | (2.3) | 291 | (2.4) | 392 | (2.4) |
| Peripheral arterial disease | 437 | (0.8) | 52 | (0.8) | 61 | (0.7) | 7 | (0.1) | 3 | (0.0) | 133 | (1.1) | 181 | (1.1) |
| Aortic aneurysm | 203 | (0.3) | 34 | (0.5) | 29 | (0.3) | 0 | (0.0) | 0 | (0.0) | 65 | (0.5) | 75 | (0.5) |
| Other cardiovascular disease | 13653 | (23.5) | 1396 | (21.8) | 1858 | (22.2) | 1908 | (29.0) | 2110 | (25.9) | 2397 | (19.7) | 3984 | (24.3) |
| **Total** | **58054** | **(100.0)** | **6400** | **(100.0)** | **8374** | **(100.0)** | **6573** | **(100.0)** | **8156** | **(100.0)** | **12152** | **(100.0)** | **16399** | **(100.0)** |
