# Supplementary material for: Increasing trends in admissions due to non-communicable diseases over 2012 to 2017: findings from three large cities in Myanmar
Source: Trop Med Health. 2020 Apr 24;48:24. doi: 10.1186/s41182-020-00209-8 (PMC7181486; doi:10.1186/s41182-020-00209-8)
Supplement: Supplementary file 4 — Additional file 4: Supplementary Table 4. Distribution of number of admissions of chronic respiratory diseases during 2012 to 2017 in three tertiary hospitals of Myanmar. [file 41182_2020_209_MOESM4_ESM.docx]

**Supplementary Table 4:** Distribution of number of admissions of chronic respiratory diseases during 2012 to 2017 in three tertiary hospitals of Myanmar

|  | **Overall** | | **2012** | | **2013** | | **2014** | | **2015** | | **2016** | | **2017** | |
| --- | --- | --- | --- | --- | --- | --- | --- | --- | --- | --- | --- | --- | --- | --- |
| **Chronic respiratory disease** | **n** | **%** | **n** | **%** | **n** | **%** | **n** | **%** | **n** | **%** | **n** | **%** | **n** | **%** |
| Chronic Obstructive Pulmonary disease | 8369 | (35.5) | 765 | (31.3) | 1137 | (37.3) | 1173 | (32.4) | 1529 | (36.2) | 1905 | (39.4) | 1860 | (34.3) |
| Asthma | 2452 | (10.4) | 274 | (11.2) | 344 | (11.3) | 453 | (12.5) | 494 | (11.7) | 446 | (9.2) | 441 | (8.1) |
| Other chronic respiratory diseases | 12780 | (54.2) | 1402 | (57.4) | 1566 | (51.4) | 1997 | (55.1) | 2200 | (52.1) | 2487 | (51.4) | 3128 | (57.6) |
| **Total** | **23601** | **(100.0)** | **2441** | **(100.0)** | **3047** | **(100.0)** | **3623** | **(100.0)** | **4223** | **(100.0)** | **4838** | **(100.0)** | **5429** | **(100.0)** |
