# Supplementary material for: Increasing trends in admissions due to non-communicable diseases over 2012 to 2017: findings from three large cities in Myanmar
Source: Trop Med Health. 2020 Apr 24;48:24. doi: 10.1186/s41182-020-00209-8 (PMC7181486; doi:10.1186/s41182-020-00209-8)
Supplement: Supplementary file 5 — Additional file 5: Supplementary Table 5. Distribution of number of admissions of diabetes during 2012 to 2017 in three tertiary hospitals of Myanmar. [file 41182_2020_209_MOESM5_ESM.docx]

**Supplementary Table 5:** Distribution of number of admissions of diabetes during 2012 to 2017 in three tertiary hospitals of Myanmar

|  | **Overall** | |  | **2012** | | **2013** | | **2014** | | **2015** | | **2016** | | **2017** | |
| --- | --- | --- | --- | --- | --- | --- | --- | --- | --- | --- | --- | --- | --- | --- | --- |
| **Diabetes mellitus** | **n** | **%** |  | **n** | **%** | **n** | **%** | **n** | **%** | **n** | **%** | **n** | **%** | **n** | **%** |
| Type 1 diabetes | 150 | (1.5) |  | 9 | (0.9) | 16 | (1.3) | 10 | (10.7) | 14 | (0.8) | 30 | (1.4) | 71 | (3.0) |
| Type 2 diabetes | 5234 | (53.9) |  | 614 | (63.7) | 688 | (57.5) | 645 | (47.7) | 853 | (50.0) | 1170 | (55.2) | 1264 | (53.3) |
| Other diabetes mellitus | 4325 | (44.5) |  | 341 | (35.4) | 492 | (41.1) | 698 | (51.6) | 840 | (49.2) | 918 | (43.3) | 1036 | (43.7) |
| **Total** | **9709** | **(100.0)** |  | **964** | **(100.0)** | **1196** | **(100.0)** | **698** | **(100.0)** | **840** | **(100.0)** | **2118** | **(100.0)** | **2371** | **(100.0)** |
